# Towards liveable cities: A review of ethnicity, public urban nature space and wellbeing

**DOI:** 10.1007/s13280-023-01871-y

**Published:** 2023-05-09

**Authors:** Isabelle D. Wolf, Gordon Waitt

**Affiliations:** 1 Australian Centre for Culture, Environment, Society and Space, School of Geography and Sustainable Communities, University of Wollongong, Northfields Ave, Wollongong, NSW 2522 Australia; 2 Centre for Ecosystem Science, University of New South Wales, Sydney, NSW 2052 Australia

**Keywords:** Blue/greenspace, Ethnic equality, Migrant, Nature, Reduced inequalities, Wellbeing nature space

## Abstract

In this review, we synthesise the results of studies that examine how the relationships between public urban nature spaces and wellbeing vary by ethnicity in cities of the Global North. We searched for articles that reported on the relationships between public urban nature spaces, ethnicity and wellbeing. We found 65 articles that met our inclusion criteria. From our review, we found positive and negative relationships between public urban nature spaces, ethnicity and wellbeing in four interrelated domains: integration/relationship building, therapy, safety and capabilities/competency building. The findings of this review inform park management by offering twelve wellbeing pathways to design urban nature spaces that are more inclusive to all residents.

## Introduction

Opportunities to experience public urban nature spaces are important for wellbeing (e.g. Hansmann et al. 2007; Hartig et al. 2014). The COVID-19 pandemic underscored this importance (Grima et al. 2020; Kleinschroth and Kowarik 2020; Ugolini et al. 2020; Venter et al. 2020; Xie et al. 2020; Liu and Wang 2021). However, there are socio-demographic inequalities in attaining wellbeing outcomes provided by public urban nature spaces (Shanahan et al. 2014). Here, we focus on how ethnicity intersects with other socio-demographics to facilitate nature-based wellbeing outcomes. This knowledge is critical to design more liveable cities for an increasingly ethnically diverse urban population of the Global North (Qadeer 2016). In this article, we respond to calls (e.g. NSW Government Architect’s Office 2017) for more ethnically inclusive nature spaces in cities by identifying wellbeing pathways for ethnically diverse public urban nature spaces, while considering various attributes of people (such as socio-demographics) and places (such as facilities).

To build pathways for more socially inclusive public nature spaces for wellbeing in ethnically diverse cities of the Global North, we are unaware of any research that systematically examined this topic. Previous reviews have emphasised various aspects of ethnic engagement with urban nature including access, use, perceptions and value of nature (Gramann 1996; Rishbeth 2001; Byrne and Wolch 2009; Gentin 2011; Jay et al. 2012; Kloek et al. 2013; Wolch et al. 2014; Ordonez-Barona 2017; Boulton et al. 2018; Tandon et al. 2018; Egerer et al. 2019). Yet none of them have explicitly focussed on wellbeing outcomes and increasing our understanding of processes of inclusion and exclusion. Similarly, although several conceptual models offer insights to ethnic engagement with urban nature (Bedimo-Rung et al. 2005; Hong and Anderson 2006; Gómez and Malega 2007; Byrne and Wolch 2009; Stodolska et al. 2011; Ordonez-Barona 2017; Stodolska et al. 2017a; Cronin-de-Chavez et al. 2019; Pham et al. 2019), none of them have either focussed on wellbeing outcomes across multiple domains/spaces or established the pathways to wellbeing outcomes. Our primary aim of this systematic review is to fill this gap, seeking to better understand how ethnicity intersects with socio-demographic categories to exclude or include from attainment of nature-based wellbeing. More specifically, we aim to consolidate all available evidence on how ethnicity modifies the relationship between wellbeing and public urban nature space.

Ethnicity is a notoriously contested and complex concept. For these reasons, we define ethnicity following the two dominant definitions found in the social sciences. In North America, the term ethnicity intersects with the concept of race that is understood in terms of physiognomic distinctiveness. This is reflected in the definition of ethnicity by the terms ‘Latinos’, ‘Asians’, ‘Hispanics’, ‘African Americans’ and ‘Caucasians’, whereas in the United Kingdom, Europe, Australia and New Zealand, the concept of ethnicity is usually conceived along the lines of ancestry and the socio-cultural distinctiveness of worldviews, values and knowledge systems (Byrne and Wolch 2009). Following the lead of Head et al., (2019, p. 400), we understand ethnic minorities as the counterpart to the “*culturally and typically numerically dominant Anglo-European ethnic majorities in places such as North America, Northern Europe and Australia*”.

There are at least four reasons that indicate the importance of considering how ethnicity intersects with other socio-demographic categories in gaining wellbeing outcomes from engagement with public urban nature spaces in cities of the Global North. First, large-scale observational studies of public urban nature spaces report on differences between ethnic ancestral groups, including anticipations, practices and experiences with nature (Gramann 1996; Rishbeth 2001; Byrne and Wolch 2009; Gentin 2011; Byrne 2012; Jay et al. 2012; Kloek et al. 2013; Wolch et al. 2014; Boulton et al. 2018; Tandon et al. 2018; Egerer et al. 2019). Second, numerous large-scale surveys suggest inequalities in attaining wellbeing benefits of public nature space along the lines of ethnicity and relatedly for class and education (Tierney et al. 2001; Stodolska et al. 2010; Kloek et al. 2013; Das et al. 2017). Third, other large-scale surveys and qualitative studies have found that public nature spaces are frequently experienced as sites of racial discrimination (Joassart-Marcelli 2010; Stodolska et al. 2010; Das et al. 2017; Xiao et al. 2018). There is a whiteness to many public nature spaces that excludes along the lines of ethnic ancestry. Fourth, a meta-analysis for national and city parks by Weber and Sultana (2013) and Byrne (2012) raised concerns around low visitation rates amongst ethnic minority groups and diminished wellbeing. Together these data suggest that the wellbeing benefits of public nature spaces vary by how ethnicity intersects with age, gender, education and class. Indeed, the majority of people visiting public nature spaces in cities of the Global North are white, earn a high annual income and possess a relatively high degree of education (e.g. McPhearson et al. 2020).

This review paper is structured in the following sections to identify wellbeing pathways to address unequal access to public urban nature space wellbeing based on ethnicity. The “Towards a more relational paradigm” section offers a theoretical background to enable our aim by integrating ideas on "outcomes-focussed management" (Driver 2008a, 2008b) from leisure science and ‘spaces of wellbeing’ (Fleuret and Atkinson 2007) from human geography. The “Methods” section outlines our approach to the literature review and analysis. The next section identifies wellbeing pathways within four domains/spaces of wellbeing that are emphasised in existing research on the relationship between ethnicity and public urban nature engagement. “Discussion, conclusions and outlooks” sums up the key findings, limitations and opens perspectives for research addressing the relationship between ethnicity, wellbeing and public urban nature.

## Towards a more relational paradigm

**Fig. 1:**
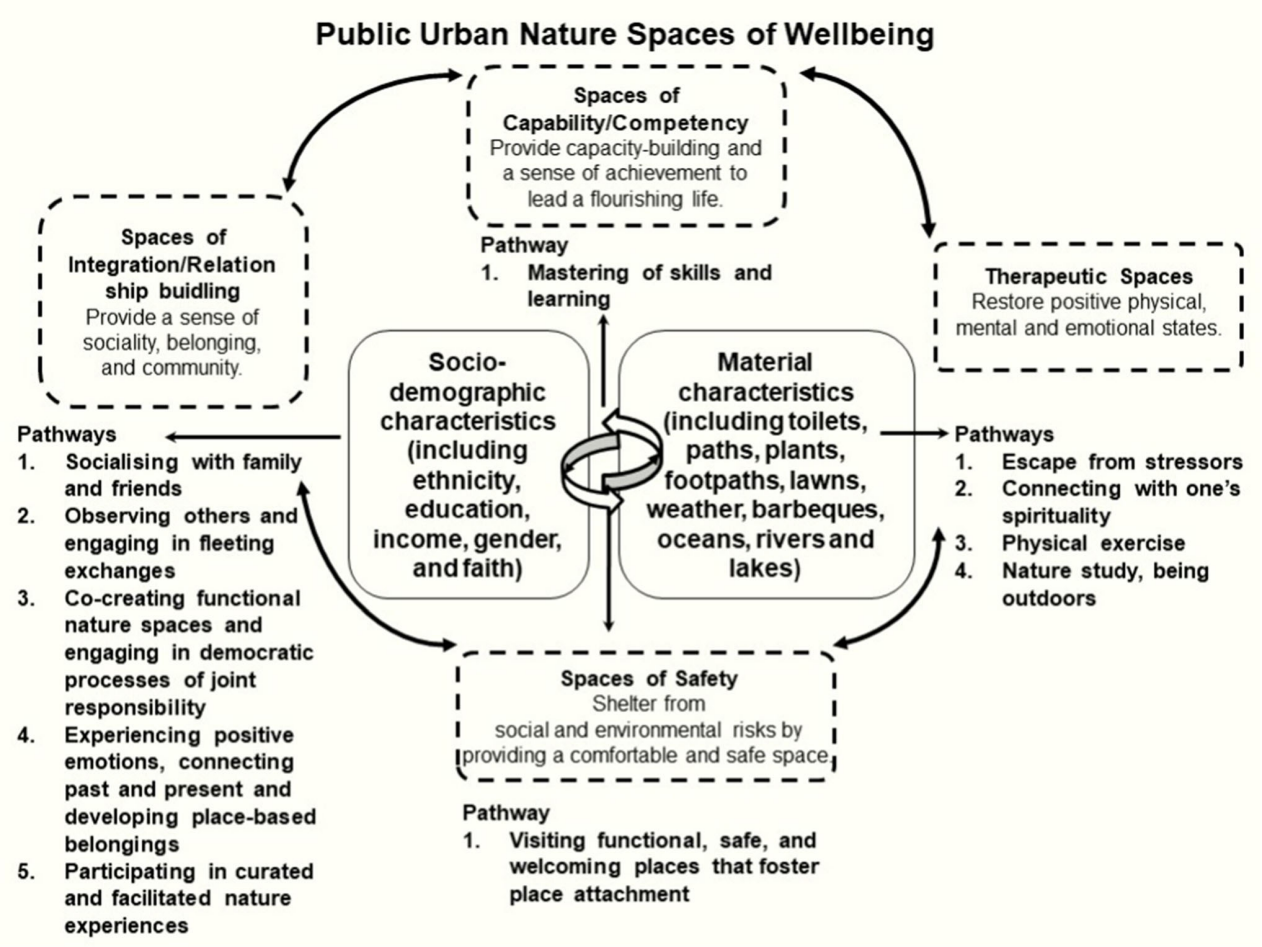
Core elements of the frameworks used for this review based on the foundational works by Driver (2008a, 2008b) on ‘outcomes-focussed management’ of nature-based recreation and Fleuret and Atkinson’s (2007) ‘spaces of wellbeing’ framework. The core elements were arranged in the form of a conceptual model titled ‘*Public Urban Nature Spaces of Wellbeing*’ showing the pathways to attain wellbeing and the moderating socio-demographic and material attributes as identified in the systematic literature review of the present study

At a theoretical level, in Fig. 1, the public urban natures spaces of wellbeing framework, illustrates how wellbeing pathways for environmental justice may be achieved in ethnically diverse cities. The public urban nature spaces of wellbeing framework melds the environmental justice dimensions of park management associated with Driver (2008a, 2008b) with a spatial approach that speaks to eudemonic and hedonic understandings of psychological wellbeing (Fleuret and Atkinson (2007). Driver (2008a, 2008b) advocates for “outcomes-focussed management” of public urban nature with the aim to optimise recreational wellbeing benefits for all by identifying inclusion pathways within multiple domains including: health, competence building and relationship building.

Fleuret and Atkinson’s (2007) ideas on spaces of wellbeing are helpful to open up new avenues for analysis for environmental justice as an embodied spatial practice. Fleuret and Atkinson’s (2007) placed-based analytical framework conceives of the ongoing dynamic human interaction with the socio-material realm as key to understanding wellbeing. They argue that their framework bridges definitions of wellbeing as either hedonic or eudemonic. A hedonic approach understands wellbeing in terms of pleasure experienced and the absence of negative emotions or moods. Emotions matter in understanding how our wellbeing is bound up with public urban nature spaces. Whereas an eudemonic approach focuses on an individual’s capacity to create wellbeing by leading a meaningful life and realising one’s potential, including learning new skills and social integration. The ‘spaces of wellbeing’ framework has been adapted in other contexts because of this capacity to transcend the hedonic and eudemonic divide, including fly-in, fly-out workers (Gorman-Murray and Bissell 2018), education (Fleuret and Prugneau 2015), art programs (Atkinson and Robson 2012) and fuel poverty (Waitt and Harada 2019).

Fleuret and Atkinson (2007) illustrate that place is far more than a backdrop or location where wellbeing occurs. Instead, wellbeing is conceived as comprised of intersecting spaces of capabilities/competencies, spaces of security, spaces of integration/relationship building and therapeutic space. For examples, spaces of capabilities/competencies are concerned with eudemonic wellbeing. The issue here is understanding how urban natures spaces are designed and facilities become taken-for-granted in policy and public discourses, and how these norms may vary socially and spatially. This includes the uneven geographical distribution of urban nature spaces in the city (distributional justice). Equally, focus is on the social construction of those who visit urban nature spaces and how these are gendered, classed and racialised (recognitional justice). Integrative spaces refer to social associations with humans and non-humans that can contribute to place-based belongings, self-esteem, competencies and ‘happiness’ or pleasure. For example, Veitch et al. (2013) illustrate how possibilities emerge for hedonic wellbeing through generating a sense of social integration and capacity building by the provision of physical activities, shade trees, bathrooms and turfgrass (procedural justice). Spaces of security enable opportunities to flourish by offering protection from social and contextual risks. For example, Lis and Iwankowski (2021) show experiences of safety are diminished by dense vegetation in city parks, restricting where women are willing to walk alone. Finally, therapeutic spaces offer possibilities for wellbeing by providing opportunities for emotional and physical relaxation from multi-sensory encounters including plants, animals, weather and water (seeing, smelling, hearing, moving and touching). For example, the strong therapeutic effects of public urban nature spaces are discussed by Berman et al. (2008), Krabbendam et al. (2021) and Lederbogen et al. (2011).

The spaces of wellbeing framework is helpful for thinking how environmental justice is bound up in not only physical proximity to public urban nature spaces (distributional justice), the absence or presence of certain facilities (procedural justice) and social norms (recognitional justice) but also in emplaced emotions, including relaxation, happiness and safety. For instance, the distribution of urban nature spaces often disproportionately benefits affluent white men (e.g. Wolch et al. 2014). Whereas procedural injustice is illustrated by when ethnic minorities stop visiting public nature spaces when they cannot relate to the rules and regulations (Mitchell and Staeheli 2005; Byrne et al. 2007). Finally, as discussed by Leikkilä et al. (2013) and Peters et al. (2016) procedural and recognitional justice requires giving ethnic minorities a greater involvement in public urban space planning processes.

At a methodological level, a spatial conceptualisation facilitates the mapping of urban public nature wellbeing spaces in a geospatial analysis program, including attributes and facilities required for a person to lead the life they value, alongside those that enable relaxation and safety. A tangible tool for public urban nature space management arises from mapping spaces of wellbeing in relation to social and material attributes alongside the socio-demographics of visitors.

## Methods

This review is part of a larger project examining the relationship between ethnicity, public urban nature spaces and wellbeing in cities of the Global North. We adopted a systematic literature review to source articles, following the process outlined by Pickering and Byrne (2014). This involved identifying key words, searching databases, assessing and selecting publications, and thematic coding of the literature to identify framework components.

We conducted a keyword search during May 2020 in five databases (Google Scholar, Web of Science, Science Direct, Scopus and ISI Web of Knowledge) (Pickering and Byrne 2014). Mindful that ethnicity is a contested concept, keywords deployed included both ‘race’ and ‘ethnicity’ alongside synonyms, including ‘ethnic (minority)’, ‘refugees’, ‘migrants’, ‘immigrants’ and ‘cultural ancestry’. These keywords were searched in various combinations with various definitions of public urban nature including: ‘blue/greenspace’, ‘blue/green space’, ‘blue/green areas’, ‘parks’, ‘nature (spending time)’, ‘outdoor (recreation)’, ‘recreational (open) spaces’, ‘leisure’, ‘free time’, ‘urban forest’, ‘natural environment’, ‘natural area’, ‘countryside’, ‘forest’, ‘urban environment’ and ‘wellbeing (benefits, outcomes, constraints, barriers)’. We terminated the search once no relevant result was found after 5 pages (Pickering, pers. com.). This stems from the notion of a reverse capture–recapture system where the likelihood to find further relevant publications decreases substantially if you do not find anything for a certain number of search results. Articles were included if they were: (1) published in English peer-reviewed academic journals; (2) analysed the potential effect of ethnicity on the relationship between public nature space and wellbeing in cities of the Global North—here ‘effect’ refers to differences in values, knowledge systems, practices and magnitude or direction of wellbeing experiences. The systematic review yielded 65 relevant articles with full text, after screening and removal of duplicates.

The selected literature covers the fields of leisure and recreation, landscape and urban planning, parks and protected areas, health, geography, environmental management, immigration studies, environmental education and environmental psychology. North American cities are the primary research focus, specifically from the United States of America (USA), with limited research originating from Canada. Further studies were from Europe, Australia and Aotearoa/New Zealand, as well as several papers conducting cross-country/continent comparisons (USA and Europe). Research within Europe was mainly from the United Kingdom (UK) followed by the Netherlands and Germany.

Pathways (mechanisms) to attain wellbeing and wellbeing themes emerging in the literature from the relationship between ethnicity and public nature spaces were identified by the authors using a combination of deductive and inductive reasoning (Pickering and Byrne 2014). This entailed a process of coding for themes through immersion in the literature. To ensure inter-rater reliability both authors coded the literature and resolved disagreements by consensus. Emerging wellbeing themes were coded into four domains/spaces based on the foundational work of Fleuret and Atkinson (2007) and Driver (2008b): social integration/relationship building, therapeutic effects, security and capacity/competency building. Next our thematic analysis coded pathways towards wellbeing achieved from the relationship between public urban nature space and ethnicity, differentiated along the lines of other socio-demographics and people attributes. Twelve pathways were identified by this analysis (Fig. 1). The thematic analysis also yielded rich data on contextual factors of public nature spaces (including facilities and crowds) that work towards or against wellbeing (Fig. 1).

## Urban public nature space, ethnicity and pathways towards wellbeing

Our systematic review identified 12 pathways that enhance the relationship between public urban nature space and wellbeing for ethnic minorities in cities of the Global North (Fig. 1). Overall, these pathways show how different wellbeing benefits for urban public nature space arise from how ethnicity intersects with other socio-demographic variables, including age, household composition, religion and gender. Moreover, these pathways demonstrate how differences in wellbeing outcomes from visiting urban nature spaces arise from how ethnicity intersects with place-based attributes such as views, shade trees, cut grass and facilities such as toilets, parking and sports fields. Here, we discuss the 12 wellbeing pathways towards ethnically inclusive public urban nature space in the four domains of our framework where they emerged most prominently. That said, pathways often sat across each of the four conceptual wellbeing domains or spaces, that is safety, therapy, integration/relationship building and capabilities/competencies. For example, place-based attachments with public urban nature spaces offers wellbeing benefits derived from both social integration and the safety of a ‘home’.

### Spaces of integration/relationship building

Wellbeing from spaces of integration/relationship building equates to needs for love and belonging satisfied through the building of friendship and family relations, developing a sense of place and experiencing love for nature (Finnis 2011). Wellbeing from spaces of integration/relationship building was achieved through a process of ‘home making’ in public nature spaces, often conveyed as a sense of belonging. This wellbeing benefit was achieved by becoming familiar with a (new) public nature space. In what follows, we discuss five pathways towards wellbeing from spaces of integration/relationship building of ethnically diverse urban communities, alongside the moderating contextual factors.

#### Socialising with family and friends

The importance of public urban nature spaces for sustaining the relationships of family and friendship of different ethnic groups is a constant theme. Public nature spaces were particularly important for the large family gatherings of ethnic minorities that relied upon food-related activities and associated facilities (Cattell et al. 2008; Stodolska et al. 2017b). For example, Whiting et al. (2017) show how picnicking by Latinos maintains social relationships with family and friends, and children. Cronan et al. (2008a, b) describe the importance of resting/sitting/relaxing activities for large, multigenerational, family-oriented groups of Latinos in the case of Lincoln Park, Chicago. These social relationships were founded on the appropriation of the park at weekends by bringing their own barbeques. Similarly, most non-Western ethnic minorities (Turks, Moroccans) in Europe favoured having a picnic or a barbecue, meeting people and playing soccer (Peters et al. 2010). The sociality of public nature experiences of ethnic minority groups contrasts with numerous studies that highlight a preference among the white ethnic majority for active individual or small-group visits to public nature spaces including walking (the dog), jogging, hiking and swimming (e.g. Gobster 2002; Tinsley et al. 2002; Ho et al. 2005; Sasidharan and Godbey 2005; Lovelock et al. 2012; Kloek et al. 2015). This may reflect different Western values that emphasise individuality and mobility (versus sociality) (Tinsley et al. 2002) and independent (versus interdependent) self-construal (Markus and Kitayama 1991).

Notwithstanding the importance of resting/sitting/relaxing activities, studies of people with an African-American ancestry highlight the importance of friendships configured by group sports such as basketball and organised activities (Floyd et al. 1994; Payne et al. 2002). People with an Asian ancestry often reported configuring social bonds through activities like walking, hiking, fishing, golf and volleyball (Gobster 2002; Payne et al. 2002; Byrne and Goodall 2013; Höglhammer et al. 2019). Regardless of activity, visitation rates for ethnic minority groups were higher amongst those who conveyed integrative wellbeing benefits, as exemplified by Turkish and Moroccan migrants in the Netherlands (Peters et al. 2016). Thus, to generate wellbeing benefits of spaces of integration/relationship building, public urban nature spaces must facilitate the social activities sought after by different people with different cultural ancestries.

#### Observing ‘others’ and engaging in fleeting exchanges

Public urban nature spaces generate possibilities for people differentiated by cultural ancestries to interact because social norms governing proximity and engagement are often suspended. Thus, for ethnic minorities, possibilities arise to develop a sense of ‘localness’ through inter-ethic interactions including micro-corporeal exchanges; that is a wave, a nod, a smile or simply saying ‘hello’ or ‘good morning’ (Peters 2010; Rishbeth and Powell 2013). Peters (2010) found that while inter-ethnic interactions in Dutch parks were rare, people from different ethnic backgrounds still appreciated being together. Furthermore, for some, observing everyday western public urban nature practices generates significant social integration wellbeing benefits. For instance, in the UK migrants from Asia and Africa were delighted by bird boxes and play equipment which were uncommon in their homelands (Rishbeth and Finney 2006). Public nature spaces that allow different ethnicities to mingle and observe may create a sense of wellbeing from social integration.

Yet, crucially, other research suggests that encounters between strangers with different cultural ancestries tend to be too fleeting (Stodolska et al. 2017b) and cursory (Jay and Schraml 2009; Seeland et al. 2009) to sustain social integration wellbeing benefits. Fleeting interactions mainly occur around conversation hooks such as sports, children, dogs and foreign accents (Peters et al. 2010). Furthermore, western social norms configure urban nature spaces for seclusion and introspection (Kloek et al. 2013). Equally, language barriers may pose another hurdle that prevents social inclusion and belonging (Peters 2010). Who ethnic minorities encounter in public nature spaces, and what these people do, impacts on the possibilities for social integration wellbeing experiences (Tinsley et al. 2002).

#### Co-creating functional public nature spaces and engaging in democratic processes of joint responsibility

Community gardens are reported in the literature as an important site of social integration wellbeing benefits from building inter-ethnic friendships (e.g. Hoffman 2019). The ties of friendship arising from community gardening are founded upon close physical proximities alongside shared responsibilities, values and work experiences (Shinew et al. 2004; Glover et al. 2005). Stodolska et al. (2017b), Horolets et al. (2019) and Peters et al. (2016) examine the creation of allotment gardens and redesigning parks to create convivial inter-ethnic public nature spaces. Allotment gardens and redesigning parks create opportunities for place-based belonging by ascribing ethnic minorities’ own meanings. However, as Rishbeth and Powell (2013) discuss, creating a ‘personal fit’ with a public nature space does not need to involve elaborate physical alterations and may simply mean setting up a picnic site (Byrne and Goodall 2013). Also, Cronan et al. (2008a, b) and Loukaitou-Sideris (1995) discuss the integrative wellbeing benefits of neighbourhood pocket parks in the USA, because they mimic the functions of the Spanish ‘plazas’. Thus, public nature spaces need to perform functions aligned with personal needs that vary according to values, worldviews and knowledge systems of different cultural ancestries. Social integration wellbeing benefits arise from the co-creating of public nature spaces like allotment gardens and plazas that generate shared responsibilities alongside reflecting the values and knowledge systems of a particular cultural ancestry.

#### Experiencing positive emotions, connecting past and present and developing place-based belongings

Our review suggests that social integration wellbeing experiences occur through engaging in public nature-based practices that emotionally connect ethnic minority groups to their ‘new’ home country by generating memories of their childhood and/or country of birth. As Virden and Walker (1999), Tolia-Kelly (2004) and Ward Thompson (2011) argue, physical sensations triggered by public nature spaces are embodied, and those experiences from childhood may help shape preferences later in life. For some, the positive emotions are evoked by similarities to their country of birth including topography, weather elements and plants (Byrne and Goodall 2013; Leikkilä et al. 2013; Rishbeth and Powell 2013). For example, Rishbeth and Finney (2006) discuss the importance of familiar plants found in Botanical Gardens and community gardens in evoking positive emotions and shaping migrants’ narratives of their destination country as home. The emotions and memories evoked by familiar topography and plants may be conceived as generating intimate relations that bridge both time and space (Jay and Schraml 2009; Goodall 2012; Coughlan and Hermes 2016; Stodolska et al. 2017b). For others, positive emotions that connect the present to the past are evoked by specific practices, like fishing, walking, or picnicking. For example, Lovelock et al. (2012) and Winter et al. (2004), discuss how some Chinese and Filipino migrants capitalise on their knowledge of fishing to build positive emotional connections and functional dependencies to their new home country.

#### Participating in curated and facilitated nature experiences

National governments may enrol public nature spaces through programs designed to help acculturate new migrants in society. Such government funded projects often employ organised outdoor activities that bring people of different cultural ancestries into physical proximity. Nature is then often positioned as a safe environment to welcome new migrants, to challenge racialised stereotypes and to ultimately provide a basis for social inclusion by facilitating contact between established communities and migrants (Morris and O’Brien 2011; Hordyk et al. 2015). Examples of officially endorsed nature-based activities include guided public urban nature tours offered to migrants in Austria (Höglhammer et al. 2019) and Sweden (Singleton 2021); structured programs for acculturation through visiting Canadian parks (Hurly and Walker 2019); tours provided in community gardens and labyrinths (Hoffman 2019), and ‘welcoming walks’ (Leikkilä et al. 2013). These programs are not a blank backdrop for social inclusion but infused with the cultural values and knowledge systems of the host nation (Peters et al. 2010; Rishbeth et al. 2019). Consequently, these programs have been criticised because of their political agendas of acculturation and whether physical proximity by bringing people together is enough to dissolve prejudice (e.g. Singleton 2021).

Opportunities for social integration wellbeing benefits have been identified through organising outdoor activities aligned with the needs of people with different cultural ancestries, especially if they were to be conducted in a variety of languages. For example, Wolf et al. (2015) illustrate how guided nature walks create opportunities for social integration. Stodolska et al. (2010) demonstrate how social integration arise from targeting specific cohorts of people with different cultural ancestries, such as families, through field trips, soccer games and dances, or mother–children walks. Whereas Cronan et al. (2008a, b) advocate for gender-specific programs that address the female underrepresentation in outdoor activities. Finally, Hordyk et al. (2015) emphasise the importance of a third party in helping migrants with different cultural ancestries to access and connect with public urban nature spaces. Migrants in this study stressed that while being in a ‘survival mode’ of adjusting to a new life and language, organising leisure trips to visit parks was otherwise considered a luxury. This research points to opportunities and constrains for wellbeing benefits derived from public urban nature as spaces of social integration through the deployment of guided nature experiences for ethnically diverse urban populations.

### Therapeutic spaces

Public nature as therapeutic spaces for ethnic minorities are widely demonstrated (Pretty et al. 2005; Maas et al. 2006). The concept of “biophilia” attributes the therapeutic outcomes to an innate desire and affiliation with the non-human world (Nisbet et al. 2011). Our literature review revealed four prominent pathways to generate therapeutic public nature spaces alive to different cultural ancestries.

#### Escaping from daily and past stressors

Public urban nature spaces are understood by most urban residents as healthier locations (Stodolska et al. 2011). Regardless of ethnicity, the therapeutic benefits of nature spaces are often evoked through the notion of ‘escape’. That said, different ethnicities played out in two important ways.

First, for some ethnic minorities therapeutic public nature spaces hold particular significance. For example, Tinsley et al. (2002) reported from a survey of park visitors in the USA that the opportunity to escape everyday responsibilities was valued even more by people classified in this study as African, Hispanic and Asian American compared to Caucasians. Likewise, Gobster (2002) reported that ethnic minorities rated specific natural attributes such as scenic views, open space, water and fresh air at least as highly if not higher than the white ethnic majority. This is especially true for those without access to nature in their domestic environment. For some new migrants or ethnic minorities, engagement with public nature space takes on particular significance if it offers respite from heightened stressful living situations, including constrained housing conditions (Hordyk et al. 2015). Equally, for some refugees, public urban nature spaces may serve a palliative function after resettlement. For example, Coughlan and Hermes (2016) reported on the palliative role of urban natures for Somali Bantu women refugees.

Second, different ethnicities played out in terms of how therapeutic wellbeing benefits were attained through preferred features of nature spaces, activities and experiences (Cronan et al. 2008a, b; Lovelock et al. 2011). Several studies reported that Caucasian park visitors tend to favour more ‘natural’ versus ‘highly managed pragmatic’ spaces (Blahna 1992; Baas et al. 1993; Gobster 2002) and find facilities at times intrusive (Kaplan and Talbot 1988). Whereas, Ho et al. (2005) reported on African-American and Hispanic park visitors as affording the highest importance ratings to recreational facilities, followed by Korean, Chinese and then Caucasian park visitors. Explanations for this difference turn to different cultural values and knowledge. For example Buijs et al. (2009) points to differences between Islamic and Christian understanding of human relationships with nature. Pathways to therapeutic wellbeing benefits through escaping the everyday requires careful consideration of how different cultural ancestries effect preferred public nature space facilities and physical attributes, along with activities and experiences, and their different living conditions.

#### Connecting with one’s spirituality

Perceived as spiritual sanctuaries, public nature spaces offer therapeutic wellbeing benefits, often aligned with different religious faiths. For example, Thomas (2002) discusses the importance of Sydney national parks in Australia as ‘forest monasteries’ for Vietnamese Buddhists in which they meditate. Goodall (2012) reported that Vietnamese and Arabic Australians were drawn to urban natures because they felt close to God.

#### Physical exercise

Physical therapeutic wellbeing benefits of public urban nature spaces through exercise tend to be the reserve of the white ethnic majority (e.g. Tinsley et al. 2002; Sasidharan and Godbey 2005; Kloek et al. 2015; Das et al. 2017). For ethnic minorities, health benefits of physical exercise in public urban nature spaces are limited to the context of group sports (K. Lovelock et al. 2011). However, there are important differences between ethnic groups (Cronan et al. 2008a, b; Lovelock et al. 2012). For example, in one study, people with African-American and Caucasian ancestry rated exercise and related therapeutic wellbeing benefits higher than those with Hispanic and Asian ancestry (Tinsley et al. 2002). Also, Stodolska et al. (2011) found that physical health benefits such as lowering blood pressure or decreasing obesity were not stated ‘directly’ by Mexican Americans. This might suggest that ‘health’ may be understood differently depending on the cultural context, and benefits may be implied by mentioning sport fields rather than stating them overtly. Cronan et al. (2008a, b), Dolash et al. (2015) and Das et al. (2017) offer explanations for differential participation rates including, lack of awareness of the health benefits (Stodolska et al. 2011), appropriate sporting facilities and gendered, raced or sexed sporting organisations. Pathways to the therapeutic wellbeing benefits of physical activity in public nature spaces require offering regular physical activities that appeal to different cultural ancestries and addressing ongoing racism, homophobia and gendered discrimination in sport.

#### Studying nature and enjoying spending time outdoors

The therapeutic benefits that accrue from studying urban nature and enjoying spending time outdoors in public urban nature spaces did not vary by ethnicity. For instance, Lovelock et al. (2012) in their study on migrants and public nature space visitation in Aotearoa/New Zealand found that the most important personal benefit by far was enjoyment of the outdoors. However, differences emerge given cultural preferences related to not only type of activities, facilities but also desired wildlife (Cronan et al. 2008a, b; Lovelock et al. 2011).

### Spaces of safety

Safety-related wellbeing addresses the human need for safety attained from the security of the body, family, property and health. Spaces of safety contribute to wellbeing where the material and social context are perceived to offer shelter from risk or chaos in everyday life. The safety of public urban nature spaces for ethnically diverse urban populations was only discussed in a handful of our selected papers. Conversely, our literature review points to how safety concerns from perceptions of nature as dangerous, poor lighting, dense vegetation, inter-ethnic conflict, alongside gendered and racialised discrimination are working against wellbeing. Nonetheless, the relationship between safety and public urban nature space is crucial.

#### Visiting functional, safe and welcoming places that foster place attachment

Loukaitou-Sideris (1995) and Rishbeth (2001) demonstrate that regardless of ethnicity, visitation to a public nature space was always underpinned by an anticipation of safety, alongside cleanliness and well maintained facilities, including the provision of parking and toilet facilities. Safe wellbeing spaces emerge when ethnic minorities develop functional place attachment to public nature spaces where they can engage in culturally desirable leisure activities (Rishbeth and Powell 2013). Knowing that places exist that fulfil needs for outdoor activities increases feelings of security.

That said, perceptions of safety in public nature spaces varied with ethnicity and gender (Golledge 1997). First, perceptions of public nature spaces as threatening, or safe, may reflect different cultural world views and values (Wallace and Witter 1992; Gramann 1996). For example, Virden and Walker (1999) reported from a survey of national park visitors in the USA that people with a Caucasian ancestry perceived forest environments to be safer than those with an African-American or Hispanic ancestry.

Second, public nature spaces are often sites of white racial privilege (J. Byrne and Wolch 2009). White privilege comes with a sense of invisibility that allows the body to access urban nature spaces unmarked. Only, when this sense of privilege is threatened does a heightened self-awareness occur. For example, Gobster (2002) and Stodolska et al. (2011) discuss how racialised spatial segregation in public urban nature spaces in the USA become the focus of inter-ethnic conflicts when territorial boundaries are traversed. Stodolska et al. (2011) discuss public urban nature spaces as sties of racial discrimination, fear, gang violence, interracial conflict and segregation discrimination. Direct discrimination was reported in the form of verbal harassment, physical gestures and assaults (Gobster 2002; Stodolska et al. 2011; J. Byrne 2012; Das et al. 2017; Waitt et al. 2021). Most frequently, people from ethnic minority groups, particularly women, reported experiences of fear from discrimination in public nature spaces (Le 2012; Das et al. 2017). Numerous authors described the discrimination of ethnic minority groups in public nature spaces by police, park staff and/or visitors, (e.g. West 1989; Blahna 1992; Baas et al. 1993; Floyd and Gramann 1995; Gobster 2002; K. Lovelock et al. 2011; Stodolska et al. 2011; Le 2012; Das et al. 2017). Whereas Le (2012) reports how people with Hispanic ancestry in public parks of the USA become the racialised ‘other’ by a lack of information and interpretive programs available in their native language.

How institutionalised racism plays out in maintaining white male privilege in urban nature spaces is discussed in the literature. For example, Bedimo-Rung et al. (2005) discuss the need for increased funding for inclusive signage alongside better lighting, patrolling and vegetation management to help create ethnically inclusive public urban nature spaces. Roberts and Chitewere (2011) point to the importance of increasing ethnic diversity among park staff.

### Spaces of capabilities/competencies

Capability-building wellbeing connects with the needs of esteem and self-actualisation and relates to confidence building, gaining respect by others, achievement, problem solving, creativity and spontaneity. Fleuret and Atkinson (2007: p. 109) describe capabilities as “*a range of attainable and valuable functionings including sets of skills and power*”... that *“provide the ability to do or be a range of things*”. They further state that the “*interest here lies in exploring the nature of settings that enable the translation of potential capabilities into attained functionings*”. Fleuret and Atkinson (2007) introduce several established capability sets which include knowledge building and the mastering of skills (Nussbaum 2001; Finnis 2011). By drawing on Sen (1992) they suggest that the material and immaterial aspects of space enhance, or constrain, wellbeing through the realisation of capabilities and, therefore, self-flourishing.

#### Mastering of skills and learning

Examples of how public urban nature spaces operate along the line of capability/competence building is how people regardless of ethnicity may master new skills such as gardening and fishing (Hoffman 2019). Community gardens are reported in the literature as important sites of capability building for new migrants. For example, Stack and Iwasaki (2009), who studied Afghan refugees who had recently migrated to Canada, reported that the cross-cultural interactions of community gardens provided opportunities for problem solving, socialisation and language learning. Indeed, community gardens function as sites of education and self-enhancement for ethnic minority groups (Tinsley et al. 2002; Sasidharan and Godbey 2005; Rishbeth and Finney 2006; Stodolska et al. 2017b).

Pathways to capability-building benefits for an ethnically diverse urban population occur through the provision of community gardens to facilitate skills, build confidence and self-esteem. Capability-building benefits are maximised when people are given a greater opportunity to be involved in the decision-making process of not only community gardens (Shinew et al. 2004; Glover et al. 2005) but municipal land use planning (Leikkilä et al. 2013; Peters et al. 2016). Critical to generating pathways to capability-building wellbeing is addressing barriers that prevent ethnic diversity in municipal land use planning and management processes around public urban nature spaces.

## Discussion, conclusions and outlooks

In increasingly ethnically diverse Global North cities, access for all to public urban nature spaces for wellbeing is a priority among policy makers. Liveability management faces the difficult challenge to not only define both ethnicity and wellbeing, but to define meaningful relationships between public urban nature spaces and the overall wellbeing of socially diverse communities. As Byrne and Wolch (2009) and Cronin-de-Chavez et al. (2019) advocate, relational geographical conceptual frameworks should be employed that are holistic and interdisciplinary enough to (i) consider the multiplicity of wellbeing outcomes that bridge eudemonic and hedonic definitions, (ii) cope with the agency of material things (trees, grass, facilities, weather), (iii) account for nature and nature-based leisure practices as always socially constituted, (iv) handle the spatial dimensions of wellbeing and (v) can manage the intersections between ethnicity, religion, household composition, gender and class. By contrast, so far, research on the relationship between urban public nature space and wellbeing has been dominated by either ‘traditional’ public health approaches that conceive of a biomedical body (for example Bedimo-Rung et al. 2005; Stodolska et al. 2011, 2017a) or the attitudes, behaviour and perception frameworks of individual participants found in psychological approaches (for example Payne et al. 2002; Gómez and Malega 2007; Pham et al. 2019). These approaches can be summarised as aspatial, fixed and individual orientated towards behaviour choices.

Even though these approaches are clearly important in responding to liveability by highlighting unequal access to urban public nature by ethnicity, they suffer from theoretical limitations that make them unable to fully address the challenge of accessing public urban nature for ethnically diverse cities. We notably argue that, by focussing on attitudes, perceptions and behaviour or on physical activity only, they (i) neglect the extent how taken-for-granted sets of ideas about ethnicity and urban nature sustain uneven access and liveability and (ii) do not consider the numerous interactions between social practices, social norms and materials that altogether define ethnicity and wellbeing as always emplaced and changing through time. For these reasons, we draw on relational thinking on offer in human geography and leisure sciences to promote alternative ways of thinking about wellbeing and identifying management pathways for inclusion.

To identify pathways for ethnic inclusion in public urban green spaces we offered a thematic analysis of the literature based on the theoretical lens of Fleuret and Atkinson’s (2007) ‘spaces of wellbeing framework’ and the foundational works by Driver (2008a, 2008b) on ‘outcomes-focussed management’ of nature-based recreation. Our thematic analysis suggested that wellbeing when conceived relationally and spatially was achieved through four intersecting domains: spaces of integration/relationship building, therapeutic spaces, spaces of safety and spaces of capability/competencies. These public urban nature spaces of wellbeing emerged through different activities of ethnic groups and in a different social and material milieu. For examples, public urban nature spaces of integration/relationship building held a great significance among ethnic minorities because they facilitated large family gatherings and food-related passive activities or team sports to socialise. In contrast, the white ethnic majority were more commonly engaged in individual and mobile activities although socialising also occurred with partners or within their immediate smaller family groups. To derive social integration wellbeing benefits from public urban nature spaces, ethnic minorities typically had a greater need for facilities in line with their activity preferences. Conversely, the white ethnic majority showed a greater preference for less managed lands and built facilities apart from trails. Therapeutic public urban nature spaces featured prominently among both the white ethnic majority and ethnic minorities. However, ethnic minorities often perceived less health and wellbeing benefits, specifically those accruing from physical exercise. Nonetheless, escape from less favourable conditions at home or work life provided nature-based therapeutic benefits. Urban public nature as spaces of safety only became possible where ethnic minorities developed place-based belonging. Often public nature spaces of safety were those that connected them to their homelands. Finally, the evidence surrounding public urban nature as spaces of capability/competencies, points to their importance in mastering of new skills and building confidence. Few differences were discussed in this context between the white ethnic majority and ethnic minorities.

We identified 12 ethnicity-related pathways to enhance the relationships between public urban nature space and wellbeing (Fig. 1). This offers critical information for land managers and policy makers who make decisions on visitor experiences, facilities, site selection and conditions that may work towards wellbeing of all urban residents. Here, we highlighted some of the differences that emerged between the white ethnic majority and ethnic minorities along the lines of preferred leisure practices and ideas of nature. At the same time, noting that there were also many commonalities such as a core set of popular activities, a shared appreciation for nature and demand for certain basic facilities. Overall, the white ethnic majority was less constrained in their visitation choices and tended to visit public urban nature spaces more frequently than some ethnic minorities (e.g. Tinsley et al. 2002; Ho et al. 2005; Parker and Green 2015; Das et al. 2017). Hence, these differences are essential targets for intervention pathways. Our results provide some evidence that government should consider funding of ethnically inclusive public urban nature space that supports integrative and therapeutic wellbeing benefits by dedicating financial resources to address institutional racism, culturally inclusive park staffing, culturally identified facilities, information and plant/animal species (Byrne 2012; Kraft et al. 2018; Cronin-de-Chavez et al. 2019). Even ‘ordinary’ nature spaces like block parks and community gardens can become a significant resource for ethnic minorities if they offer safety and provision of the right facilities (e.g. Egerer et al. 2019). Coupled with our finding regarding safety and capacity-building wellbeing benefits it seems wise to invest in the provision of lighting, community gardens/allotments, involvement in neighbourhood decision-making, multi-lingual signage and guided tours.

These conclusions were reached by how the review consolidated the available evidence on the relationships between ethnicity, public urban nature and wellbeing. This review has several strengths. We conducted an extensive keyword search in numerous scientific databases. We draw on the collective evidence from 65 articles to show how the wellbeing benefits derived from public urban nature space intersect across the domains of integration/relationship building, therapy, safety and capability/competency. We advanced a conceptual framework to guide our research aim and interpret our results. That said, we acknowledge that this review also has limitations. Although our keyword search captured two dominant definitions of ethnicity, a finer keyword search may offer a more nuanced insight, for example terms conventionally associated with the concept of race like ‘diet,’religion’ and language.

Our review suggests five priorities for a future research agenda on better understanding the relationship between ethnicity, wellbeing and public urban nature spaces in cities of the Global North. First, future research is required in Australia, Aotearoa/ New Zealand, Europe, Scandinavia and the United Kingdom to address the current North American focus. Second, as acknowledged by Driver (2008a, 2008b), research is required to better understand how to implement and monitor, management pathways to enhance the connections between ethnicity, wellbeing and public urban nature. For instance, little research exists on how to create guided or facilitated experiences for ethnic minorities to enhance the public urban nature space-wellbeing relationship (Wolf et al. 2015). Third, existing nature-based integration research points to how participating in outdoor recreational activities is often coupled with environmental citizenship, tied to ideas of wellbeing, place attachment and acculturation (see Pitkänen et al. 2017; Gentin et al 2019; Singleton 2021). While outdoor leisure activities are regarded as causally powerful, their relation to wellbeing, and questions of moral and human flourishing to date has been ignored. Research is needed that investigates the implications of nature-based integration for migrant’s moral status (that is if a program is compulsory or voluntary) and moral agency (that is the right to be in an urban nature public space, though not like the white or wealthy ethnic majority). Our urban nature spaces wellbeing framework offers a capabilities approach to understanding human flourishing and moral agency by focussing on the intersections between therapeutic, safety, sociality and capability space. Fourth, our analysis focussed on differences between ethnic groups and could be expanded in the future to explore the diversity and complexity within groups of people who identify with a particular ancestry or cultural heritage. Finally, our analysis is primarily theoretical and calls for applied research. To this aim, participatory geographic information systems and geospatial analysis could facilitate an understanding of spatial inequalities in access to public urban nature spaces of wellbeing. Geospatial analysis enables the mapping of the four public urban nature spaces of wellbeing as spatial layers. Expected benefits in operational contexts may include assessing and mitigating the uneven access to urban public nature for wellbeing by ethnicity.
